# Dual thrombolytic therapy with mutant pro-urokinase and small bolus alteplase for ischemic stroke (DUMAS): study protocol for a multicenter randomized controlled phase II trial

**DOI:** 10.1186/s13063-022-06596-z

**Published:** 2022-08-09

**Authors:** Nadinda A. M. van der Ende, Bob Roozenbeek, Lucas E. M. Smagge, Sven P. R. Luijten, Leo A. M. Aerden, Petra Kraayeveld, Ido R. van den Wijngaard, Geert J. Lycklama à Nijeholt, Heleen M. den Hertog, H. Zwenneke Flach, Alexis C. Wallace, Victor Gurewich, Gregory J. del Zoppo, William J. Meurer, Hester F. Lingsma, Aad van der Lugt, Diederik W. J. Dippel, on behalf of the DUMAS Investigators, Diederik Dippel, Aad van der Lugt, Nadinda van der Ende, Bob Roozenbeek, Moniek de Maat, Leo Aerden, Ido van den Wijngaard, Heleen den Hertog, Petra Kraayeveld, Geert Lycklama a Nijeholt, Zwenneke Flach, Michael Hill, Jeremy Rempel, Ann Lowe, Hester Lingsma, Daan Nieboer, Gregory del Zoppo, Dingeman Rijken, Adam Cohen, Victor Gurewich, Aad van der Lugt, Lucas Smagge, Martin Sterrenberg, Naziha El Ghannouti, Debby Priem, Monique Batenburg, Eva Ponjee, Rieke Eilander, Joke de Meris, Tamara Dofferhoff-Vermeulen, Sanne den Hartog, Stijn Kremer, Leontien Heiligers, Angela Lansbergen-Engel

**Affiliations:** 1 Department of Neurology, Erasmus MC University Medical Center, Rotterdam, the Netherlands; 2 Department of Radiology and Nuclear Medicine, Erasmus MC University Medical Center, Rotterdam, the Netherlands; 3 Department of Neurology, Reinier de Graaf, Delft, the Netherlands; 4 Department of Radiology and Nuclear Medicine, Reinier de Graaf, Delft, the Netherlands; 5 Department of Neurology, Haaglanden Medical Center, The Hague, the Netherlands; 6 Department of Radiology and Nuclear Medicine, Haaglanden Medical Center, The Hague, the Netherlands; 7 Department of Neurology, Isala klinieken, Zwolle, the Netherlands; 8 Department of Radiology and Nuclear Medicine, Isala klinieken, Zwolle, the Netherlands; 9 Thrombolytic Science, Cambridge, MA USA; 10 Department of Medicine, Mount Auburn Hospital, Harvard Medical School, Boston, MA USA; 11 Department of Medicine, Division of Hematology, University of Washington School of Medicine, Seattle, WA USA; 12 Department of Neurology, Division of Hematology, University of Washington School of Medicine, Seattle, WA USA; 13 Departments of Neurology, University of Michigan Medical School, Ann Arbor, MI USA; 14 Departments of Emergency Medicine, University of Michigan Medical School, Ann Arbor, MI USA; 15 Berry Consultants, Austin, TX USA; 16 Department of Public Health, Erasmus MC University Medical Center, Rotterdam, the Netherlands

**Keywords:** Thrombolytic treatment, Mutant pro-urokinase, Alteplase, Ischemic stroke, Randomized controlled trial

## Abstract

**Background:**

The effectiveness of alteplase for ischemic stroke treatment is limited, partly due to the occurrence of intracranial and extracranial hemorrhage. Mutant pro-urokinase (m-proUK) does not deplete fibrinogen and lyses fibrin only after induction with alteplase. Therefore, this treatment has the potential to be safer and more efficacious than treatment with alteplase alone. The aim of this study is to assess the safety and efficacy of thrombolytic treatment consisting of a small bolus alteplase followed by m-proUK compared with standard thrombolytic treatment with alteplase in patients presenting with ischemic stroke.

**Methods:**

DUMAS is a multicenter, phase II trial with a prospective randomized open-label blinded end-point (PROBE) design, and an adaptive design for dose optimization. Patients with ischemic stroke, who meet the criteria for treatment with intravenous (IV) alteplase can be included. Patients eligible for endovascular thrombectomy are excluded. Patients are randomly assigned (1:1) to receive a bolus of IV alteplase (5mg) followed by a continuous IV infusion of m-proUK (40 mg/h during 60 min) or usual care with alteplase (0.9 mg/kg). Depending on the results of interim analyses, the dose of m-proUK may be revised to a lower dose (30 mg/h during 60 min) or a higher dose (50 mg/h during 60 min). We aim to include 200 patients with a final diagnosis of ischemic stroke. The primary outcome is any post-intervention intracranial hemorrhage (ICH) on neuroimaging at 24 h according to the Heidelberg Bleeding Classification, analyzed with binary logistic regression. Efficacy outcomes include stroke severity measured with the National Institutes of Health Stroke Scale (NIHSS) at 24 h and 5–7 days, score on the modified Rankin scale (mRS) assessed at 30 days, change (pre-treatment vs. post-treatment) in abnormal perfusion volume, and blood biomarkers of thrombolysis at 24 h. Secondary safety endpoints include symptomatic intracranial hemorrhage, death, and major extracranial hemorrhage. This trial will use a deferred consent procedure.

**Discussion:**

When dual thrombolytic therapy with a small bolus alteplase and m-proUK shows the anticipated effect on the outcome, this will lead to a 13% absolute reduction in the occurrence of ICH in patients with ischemic stroke.

**Trial registration:**

NL7409 (November 26, 2018)/NCT04256473 (February 5, 2020)

**Supplementary Information:**

The online version contains supplementary material available at 10.1186/s13063-022-06596-z.

## Background

Currently, recombinant tissue plasminogen activator alteplase is the only FDA-approved agent for the thrombolytic treatment of ischemic stroke. This thrombolytic treatment with alteplase in patients with ischemic stroke leads on average to improved reperfusion in about 30% of patients and increases the likelihood of a good clinical outcome in one of every ten treated patients [1]. Apart from its limited efficacy, it carries a risk of symptomatic intracranial hemorrhage of 6-7% [2].

Treatment with endovascular thrombectomy is effective in patients with ischemic stroke due to a large-vessel occlusion in the anterior circulation [3], which is present in at most 30% of ischemic stroke patients presenting at the emergency department [4, 5]. For patients with ischemic stroke without a large-vessel occlusion, reperfusion can only be reached through thrombolytic treatment [6]. There is a need for a better and safer thrombolytic therapy, which expands the number of patients that can be treated safely and successfully. Preclinical and clinical studies have indicated that dual thrombolytic therapy, mimicking the physiological design of thrombolysis, with small bolus alteplase followed by a mutant pro-urokinase (m-proUK) has a significant potential to be safer and more efficacious than the FDA-approved regimen of standard dose alteplase alone (0.9 mg/kg) [7–10]. M-proUK is a single-point mutation of the single-chain zymogen plasminogen activator, pro-urokinase, with less susceptibility to non-specific activation into its enzymatic, two-chain form, urokinase [9]. Moreover, m-proUK by itself does not lyse hemostatic fibrin, only partially degraded fibrin [8, 11]. When the low dose alteplase is cleared from the systemic circulation, m-proUK will only induce intravascular clot lysis, and spare hemostatic fibrin. Therefore, this therapeutic regimen has the potential to be safer and more efficacious than treatment with alteplase [7–9].

### Objectives

1. To assess the safety of treatment with a dual plasminogen activator (in short: thrombolytic), which consists of a small bolus of intravenous (IV) alteplase followed by IV infusion of m-proUK against usual treatment with IV alteplase in patients presenting with ischemic stroke.
2. To assess the preliminary efficacy of treatment with a dual plasminogen activator, which consists of a small bolus of IV alteplase followed by IV infusion of m-proUK against usual treatment with IV alteplase in patients presenting with ischemic stroke.

## Methods

### Study design

**Fig. 1:**
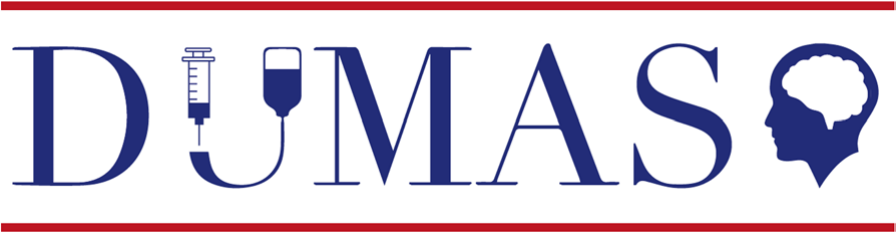
Trial logo

**Fig. 2:**
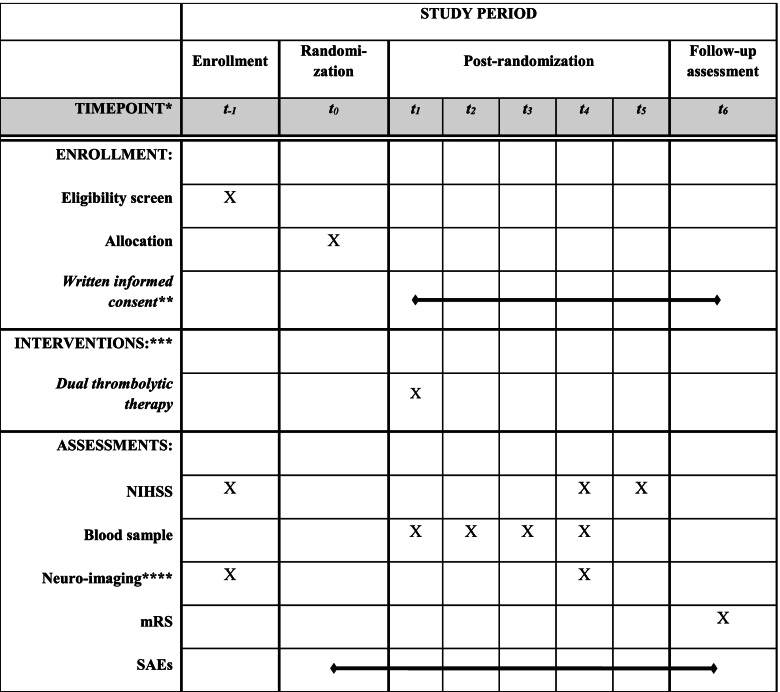
SPIRIT figure. mRS indicates modified Rankin scale; NIHSS, National Institutes of Health Stroke Scale; SAE, serious adverse events. *t_−1_: directly before randomization, t_0_: randomization, t_1_ = directly after randomization; t_2_ = at 1 h; t_3_ = at 3 h; t_4_ = at 24 h; t_5_ = at 5–7 days or at discharge if earlier; t_6_ = at 30 days. **Informed consent: as early as deemed possible after IV thrombolysis. ***Administration of IV thrombolytic therapy directly after randomization. ****Neuro-imaging at baseline consists of non-contrast CT, CT-perfusion, and CT-angiography or brain MRI and MRA. Neuro-imaging at 24 h consists preferably of a brain MRI

The DUal thrombolytic treatment with Mutant pro-urokinase and small bolus Alteplase for ischemic Stroke (DUMAS) trial is a multicenter, phase II trial with a prospective randomized open-label blinded end-point (PROBE) design and an adaptive design for dose optimization (Fig. 1). Thrombolytic treatment consisting of a small bolus of IV alteplase and IV m-proUK will be compared with usual thrombolytic treatment with alteplase 0.9 mg/kg. An overview of the main study procedures is provided in Fig. 2. Because the exact optimal dose of IV m-proUK for patients with ischemic stroke is still unknown, sequential interim analyses will be performed allowing adaptation of the IV m-proUK dose. A detailed description of this adaptive design can be found in Supplemental File 1. The “Standard Protocol Items: Recommendations for Interventional Trials” (SPIRIT) checklist is provided as Supplemental File 2 [12]. The study will run in several hospitals in the Netherlands.

### Patient population

The study population will be drawn from patients with a clinical diagnosis of ischemic stroke at the Emergency Department.

Patients are eligible for inclusion in DUMAS when they are 18 years or older and have a clinical diagnosis of ischemic stroke with a deficit on the National Institutes of Health Stroke Scale (NIHSS) of at least 1 point. Non-contrast computed tomography (NCCT) or magnetic resonance imaging (MRI) should rule out intracranial hemorrhage (ICH) and patients should meet the criteria for standard treatment with IV alteplase according to national guidelines [13]. Treatment should therefore be possible within 4.5 h from symptom onset or last seen well or between 4.5 and 12 h from symptom onset or last seen well, if the infarct core is less than 25 mL and the penumbra is at least the same size as the infarct core (i.e., total ischemic volume/infarct core mismatch ≥ 2.0) [14], or in case of lacunar syndrome [15], if there is a diffusion-weighted imaging (DWI) and fluid attenuation inversion recovery (FLAIR) mismatch, consistent with Dutch national guidelines for stroke [16].

Exclusion criteria are eligibility for endovascular thrombectomy (i.e., patients with a proximal large artery occlusion on CT angiography or magnetic resonance angiography), contra-indication for treatment with IV alteplase according to national guidelines, pre-stroke modified Rankin Scale (mRS) score > 2, known pregnancy, contra-indication for MRI, and participation in any therapeutic trial other than DUMAS.

There are no dropout criteria.

### Randomization

#### Sequence generation

Patients will be randomized 1:1 to standard thrombolytic treatment with alteplase alone vs. dual thrombolytic treatment with a bolus alteplase and m-proUK. The randomization procedure will be web-based, using permuted blocks. Randomization will be stratified for the center. The allocation sequence was generated by the independent trial statistician.

#### Allocation concealment mechanism

The allocation sequence was unknown to all investigators.

#### Implementation

Patients will be randomized by the treating physician.

### Blinding

Clinical outcomes, such as NIHSS scores and serious adverse events are collected by trained research personnel. Standardized telephone interviews to assess the mRS score at 1 month will be conducted from a central location by experienced research nurses, unaware of treatment allocation [17, 18]. They will instruct patients or relatives before starting the interview not to say anything about the performed procedure or the admission in the hospital. Neuroimaging will be assessed by an imaging core laboratory blinded to study treatment allocation. Members of the imaging core laboratory are unaware of clinical data including treatment allocation, but were informed about baseline clinical symptoms. Clinical symptoms were defined as side of the hemiparesis, presence of aphasia, or non-localizing symptoms for the patients without hemiparesis or aphasia. Follow-up neuroimaging on which the primary outcome is determined will be assessed by two independent members of the imaging core laboratory. If the two imaging core laboratory assessments do not match, disagreements will be resolved by consensus. Outcome data will be entered in a database that is kept separated from the main clinical database, which includes information about the treatment allocation.

### Study treatment

The intervention arm will receive a small bolus of IV alteplase 5 mg, which will be followed by a continuous infusion of m-proUK, either 40 mg in 60 min (initial dose) or an alternate dose, independent of patient weight. Depending on the result of interim analyses, the m-proUK dosage may be revised to:

- Higher than the initial dose, by 25% (i.e., 50 mg in 60 min)
- Lower than the initial dose, by 25% (i.e., 30 mg in 60 min)

A detailed description of this adaptive design for dose optimization can be found in Supplemental File 1. In case of a dose change, only one switch back to the original dose is allowed without further changes to the dose. The total number of different dosages used in the trial will therefore not exceed two, in order to retain sufficient precision in the estimate of dose-related treatment effect.

The control arm will receive standard treatment with IV alteplase alone in a dose of 0.9 mg/kg (10% bolus + 90% infusion in 60 min), maximum dose 90 mg.

### Study procedures

Patients undergo assessment of the NIHSS at baseline, 24 h, and 5–7 days (or discharge if earlier), which is a routine clinical procedure. It will be carried out by NIHSS-certified physicians. All patients will undergo NCCT, CTA and CT perfusion or MRI and MRA of the brain at baseline, as part of routine clinical care. CT-perfusion will be focused on the anterior circulation or posterior circulation depending on the suspected location of the ischemic stroke as determined by the treating physician. For follow-up imaging, all patients will undergo an MRI of the brain at 24 h (range: 12 to 48 h). The MRI scan will include the following sequences: [1] Susceptibility weighted imaging (SWI), [2] DWI/apparent diffusion coefficient (ADC), [3] dynamic susceptibility contrast MRI (DSC-MRI) or arterial spin labeling (ASL), [4] T2 weighted imaging (T2w), [5] T2-FLAIR. In case of any contra-indication for MRI after randomization (e.g., because the contra-indication was not known at the time of inclusion or the patient has a new contra-indication due to an intervention during hospital admission), a follow-up NCCT and CT-perfusion at 24 h will be performed instead. Blood samples will be taken at baseline, one tube EDTA (+/− 5 mL), one tube without anticoagulant (+/− 7mL) and two tubes with citrated blood (2.7 mL) will be drawn. Additional blood samples will be taken (two tubes citrated blood of 2.7 mL) at 1 h, at 3 h, and at 24 h post treatment. Biomaterials will only be collected for patients in two participating centers. Plasma samples will be stored at −80 °C for later analysis. A schedule of all activities is shown in Fig. 2.

### Informed consent

This study uses a deferred informed consent procedure, because DUMAS evaluates an acute intervention in an emergency situation concerning a life-threatening disorder [19]. Consent will be asked as early as possible, and as deemed appropriate according to the treating physician.

If a patient or his/her representative refuses to provide consent, participation in the trial will be terminated immediately. The procedure requires that all information on patients who did not provide consent is discarded and deleted. This may be against the interest of patients who did provide consent, and against the interest of the general public, as patients with (serious) adverse events might be more likely to refuse consent for participation. Not considering these records might very well result in an underestimation of the true safety and validity of the data, and it might lead to undetected safety concerns for all consenting patients in the trial. To overcome this concern, all randomized patients will be registered in an anonymized safety registry providing information on in-hospital symptomatic ICH and in-hospital mortality (both important safety variables for the study). All other information will be completely erased from the patient’s study record in case no consent is provided.

When a patient has died before consent has been obtained, their representative will be informed about trial participation. These patients will be included in all analyses, there is no opt-out option since that may bias study results.

### Study outcomes

The primary outcome is any post-intervention ICH confirmed by neuroimaging according to the Heidelberg Bleeding Classification at 24 h (range: 12 to 48 h) after study drug administration preferably by MRI (SWI) [20]. Secondary clinical outcomes are the NIHSS score at 24 h, the NIHSS score 5–7 days (or discharge if earlier), and the mRS at 30 days [21, 22]. Secondary imaging outcomes are change (pre-treatment vs. post-treatment) in abnormal perfusion volume and infarct volume at 24 h post-treatment. Secondary blood biomarker outcomes include fibrinogen, plasminogen, alpha2-antiplasmine, and d-dimers at 1 h, at 3 h, and at 24 h. Safety outcomes include symptomatic ICH according to the Heidelberg Bleeding Classification, death from any cause within 30 days, and major extracranial hemorrhage according to the ISTH criteria within 24 h of thrombolytic treatment [20, 23].

### (Serious) adverse event reporting

Safety is an issue of concern as thrombolytic treatment has a bleeding risk. Adverse events are defined as any undesirable experience occurring to a subject during the study, whether or not considered related to the investigational product. All adverse events reported spontaneously by the subject or observed by the investigator or his staff will be recorded. A serious adverse event is any untoward medical occurrence or effect that results in death, is life-threatening (at the time of the event), requires hospitalization or prolongation of existing inpatients’ hospitalization, results in persistent or significant disability or incapacity, is a congenital anomaly or birth defect, that required medical or surgical intervention, or any other important medical event that did not result in any of the outcomes listed above due to medical or surgical intervention but could have been based upon appropriate medical judgment. We will report SAEs that occurred within the follow-up period defined by the last follow-up contact.

### Data Safety Monitoring Board

The Data Safety Monitoring Board (DSMB) consists of a neurologist (chair), a neuro-radiologist, and a hematologist. An independent statistician will combine clinical and outcome data in order to report to the DSMB. The DSMB will advise the chairman of the steering committee if, in their view, the randomized comparisons have provided both (i) “proof beyond reasonable doubt” that for all, or some, the treatment is clearly indicated or clearly contra-indicated and (ii) evidence that might reasonably be expected to materially influence future patient management. Appropriate criteria of proof beyond reasonable doubt cannot be specified precisely, but the DMSB will work on the principle that a difference of at least 3 standard errors in an interim analysis of a major outcome event (e.g., any ICH, death) may be needed to justify halting, or modifying a study before the planned completed recruitment. Safety interim analyses will also include measures of efficacy (NIHSS and mRS) and are planned after the inclusion of 20, 30, 40, and 50 patients and after that with increments of 50, after the start of the trial, and after any dose change, until the trial is completed, unless the DSMB advises otherwise during the conduct of the trial. Following a report from the DMSB, the steering committee will decide whether to modify entry to the study (or seek extra data) and inform the sponsor. Unless this happens, however, the steering committee, the collaborators, and central administrative staff will remain ignorant of these analyses and results.

Apart from these safety and efficacy reports, the DSMB will receive additional analyses from an independent statistician, who will inform the DSMB on the likelihood of success or failure of the study to reach a positive result. This information will be used to advise the steering committee to adapt the dosing in the study according to pre-specified criteria as explained in detail in Supplemental File 1. The information provided in these interim analyses will not be used to discontinue the study for expected futility, as it is the intention of the steering committee to run the trial until 200 patients with a final diagnosis of ischemic stroke have been included, as long as there are no safety or efficacy concerns.

### Data collection and management

All data will be entered into a web-based trial management system (OpenClinica), which allows for edit and audit trails, by trained local research personnel. Patient records are coded by a unique study number. The local investigators will keep a list showing codes and names. Unique documents with identifying information will be stored separately from the study database in digital files, categorized by study number on a secure drive system, and only accessible to the study coordinators.

This trial qualifies as a moderate risk study, i.e., a study with a small risk of serious adverse events compared to standard treatment. According to Dutch standards, 25% of local data should be reviewed against source data. However, as this is a phase 2 trial, we will monitor a pre-specified list of local data, which includes secondary outcomes (i.e., the NIHSS at 24 h, the NIHSS at 5–7 days, and the mRS at 30 days), in 100% of the patients.

### Statistical analyses

Baseline data by treatment allocation will be reported with standard statistical procedures and missing values will be reported. For regression analyses, missing values, except the primary outcome, will be imputed using multiple imputation (*n*=5). We will perform and report 4 analyses, of which the first is the primary:

1. Simple modified intention-to-treat analysis to assess overall safety and efficacy. This is a modified intention-to-treat analysis because we exclude patients who did not give consent to participate in the study. We will additionally report safety parameters based on the full cohort, including patients who did not give consent.
2. Targeted modified intention-to-treat analysis excluding patients with a final diagnosis other than ischemic stroke to assess safety and efficacy in the target population.
3. Targeted modified on-treatment analysis to assess the safety and efficacy in patients who actually received treatment excluding patients with a final diagnosis other than ischemic stroke.
4. Per-protocol analysis.

Additionally, we will perform subgroup analyses on categorized baseline variables including age, sex, systolic blood pressure, ASPECTS, time from onset to study treatment, NIHSS score, extracranial carotid or vertebral arterial occlusion, pre-study antiplatelet treatment, DWI lesion (yes/no), and lacunar syndrome (yes/no). Subgroup analyses will be done by testing for interaction of the subgroup indicator with treatment.

The effect of the study treatment on the primary outcome will be assessed with multivariable logistic regression with study treatment as a binary independent variable (m-proUK vs. control). Adjusted and unadjusted effect estimates with corresponding 95% confidence interval (CI) will be reported. The effect estimate will be adjusted for important prognostic factors at baseline, which include at least age and time from onset of symptoms to randomization. Stroke severity (NIHSS score), lacunar syndrome (yes/no) [11], systolic blood pressure, antiplatelet treatment, and endovascular treatment (yes/no) will be considered additionally in this order. Whether the dosing (initial vs. modified) of the study treatment modifies the treatment effect, will be analyzed with a multiplicative interaction parameter in the main analysis.

The effect of the study treatment on the secondary outcomes will be assessed with multivariable linear, logistic, or ordinal regression models with study treatment as a binary independent variable (m-proUK vs. control). The effect parameter will be either a beta or (common) OR with 95% CI. This effect will be adjusted with variables that are predictive of the specific outcome measure.

The most up-to-date statistical analysis plan can be found on the website (https://dumas-trial.nl/).

### Sample size

We will include 200 patients with a final diagnosis of ischemic stroke randomized 1:1 to either standard thrombolytic treatment or dual thrombolytic treatment. We assume that the primary outcome, any ICH, will occur with a probability of 20% with standard thrombolytic treatment and a probability of 7% in the patients treated with dual thrombolytic therapy [24]. This leads to an overall effect (odds ratio (OR)) of 0.3. This sample size will provide us with a power of at least 77% to detect a statistically significant effect on the primary outcome. This estimate does not take into account the use of multivariable adjustment for differences in baseline characteristics in the primary analysis.

To ensure sufficient power in the targeted modified on-treatment analysis, an additional patient will be randomized and included (i.e., replaced) for each patient who

- did not give consent for participation in the study, or
- for any reason did not receive the full dose of thrombolytics as assigned, or
- had a final diagnosis other than ischemic stroke (i.e., stroke mimic).
  - ◦ We estimate that up to 20% of the included patients will not have a final diagnosis of ischemic stroke [25].

### Study organization

The sponsor of the trial is the Erasmus MC University Medical Center, Doctor Molewaterplein 40, 3015 GD Rotterdam, Rotterdam, The Netherlands. The steering committee is responsible for the overall supervision of the trial. Additionally, the steering committee will discuss all patients about whom doubt exists concerning the discharge diagnosis of ischemic stroke or not (i.e., stroke mimic). Every local principal investigator can propose cases for discussion. This concerns at least patients without a DWI lesion on follow-up MRI. The executive committee keeps track of trial progress and makes the strategic decisions on a weekly basis. See the “Acknowledgements” section for a full list of the investigators.

### Ethics approval

The study will be conducted according to the principles of the Declaration of Helsinki (7th revision, Brazil, October 2013), Good Clinical Practice, the Dutch Medical Research Involving Human Subjects Act (WMO), and when it becomes applicable in accordance with regulations of other countries with participating centers. The DUMAS research protocol has been approved by the Institutional Review Board of the Erasmus MC University Medical Center (MEC-2019-0001). The current manuscript is based on protocol version 1.3.2 (April 5, 2022). The most up-to-date approved trial protocol can be found on the website (https://dumas-trial.nl/).

## Discussion

DUMAS will assess the safety and preliminary efficacy of a dual acute thrombolytic treatment consisting of a small bolus of intravenous (IV) alteplase followed by IV infusion of m-proUK against usual treatment with IV alteplase in patients presenting acutely with ischemic stroke. We hypothesize that dual thrombolytic therapy with a bolus alteplase and m-proUK will reduce the occurrence of any ICH in patients with ischemic stroke compared to patients treated with alteplase alone.

### Other ongoing trials

There are currently no other ongoing trials investigating the safety and efficacy of thrombolytic treatment with m-proUK.

There are other ongoing trials evaluating the effect of a different thrombolytic agent, tenecteplase. Tenecteplase at a dose of 0.25 mg/kg is a promising alternative to alteplase, due to its ease of administration, but until now, superiority or even non-inferiority has not been convincingly demonstrated. Also, the rates of intracranial hemorrhage in patients treated with tenecteplase and alteplase are similar [25–29].

### Limitations and concerns

As DUMAS is the first randomized clinical trial to evaluate the safety and preliminary efficacy of thrombolytic treatment with m-proUK in patients with ischemic stroke, there is no clear evidence on the most effective and safe dose of m-proUK. The m-proUK dose is based on a thrombolytic treatment with alteplase and pro-urokinase for acute myocardial infarction [10]. Previous trials that evaluated thrombolytic treatment for ischemic stroke in doses similar to those used for treatment of myocardial infarction reported high rates of ICH and no beneficial effect of treatment on functional outcome [30, 31]. That prompted investigators of thrombolytic therapy for ischemic stroke to use doses of 60% to 90% of the dose used in myocardial infarction. For example, in GUSTO, a randomized controlled trial in patients with myocardial infarction, the most effective thrombolytic regimen was accelerated tPA in a bolus of 15 mg, 0.75 mg/kg in 30 min, not to exceed 50 mg, and 0.5 mg/kg, up to 35 mg, over the next 60 min combined with intravenous heparin. This means that an average patient, weighing 75 kg, would receive a total of 100 mg alteplase (the maximum dose) [32]. The total dose used in the effective landmark alteplase trials for ischemic stroke was 0.9 mg/kg, including a 10% bolus, based on safety considerations and efficacy [2]. An average patient, weighting 75 kg, would receive a total of 67.5 mg, which comes down to 67.5% of the GUSTO dose in an average person [2, 33]. Considering the increased risk of ICH after thrombolytic treatment in patients with ischemic stroke compared with patients with myocardial infarction, the cumulative dose of pro-urokinase is reduced with 33% compared to the pro-urokinase dose given to patients with myocardial infarction [10]. Because the exact optimal dose of IV m-proUK in patients with ischemic stroke is still unknown, sequential interim analyses will be performed allowing adaptation of the IV m-proUK dose.

DUMAS excludes patients who are candidates for endovascular thrombectomy (i.e., patients with a proximal intracranial large artery occlusion on CTA or MRA) due to logistics with regard to other ongoing trials in this patient population in the Netherlands, and because subsequent thrombectomy will disturb the assessment of the intervention effect. However, because DUMAS allows inclusion of all other types of ischemic stroke including lacunar infarcts, cortical infarcts, and posterior circulation strokes, we expect that results will be generalizable to all patients with an indication for treatment with IV thrombolytics.

Dual thrombolytic treatment with a small bolus alteplase followed by m-proUK has the potential to be safer than standard treatment with alteplase alone, because m-proUK by itself cannot lyse hemostatic fibrin. When alteplase is cleared from the systemic circulation, hemostatic fibrin in new lesions in the ischemic region will remain intact. Therefore, this dual thrombolytic treatment is likely to reduce the occurrence of any post-treatment ICH. ICHs can be classified as either symptomatic or asymptomatic. Several classifications are in use, but symptomatic ICH is often defined as an increase in neurological deficit of 4 points or more on the NIHSS, or death, with hemorrhage confirmed by neuro-imaging. This implies that several hemorrhages may cause more subtle deterioration and are classified as asymptomatic. Moreover, it has been suggested that ICHs classified as asymptomatic are associated with worse functional outcome and should be considered relevant for functional outcome [34–36]. These considerations argue for using all intracranial hemorrhages as the primary outcome.

Moving forward to a phase 3 trial investigation, the safety and efficacy of treatment with m-proUK should be considered in case of statistically significant treatment effect on the primary outcome (i.e., any intracranial hemorrhage) or if the estimated effect is not statistically significant, but large, consistent over secondary outcomes, and in line with the pathophysiological properties of m-proUK and the experimental studies.

## Conclusion

DUMAS is a phase II clinical trial with PROBE design, which investigates the safety and preliminary efficacy of dual thrombolytic treatment consisting of a small bolus alteplase followed by m-proUK IV infusion against usual treatment with alteplase in patients presenting with ischemic stroke. When this dual thrombolytic therapy with a small bolus alteplase and m-proUK shows the anticipated effect on outcome, this will lead to a 13% absolute reduction in the occurrence of ICH in patients with ischemic stroke.

### Trial status

Approval of the DUMAS trial protocol by the ethical board was given on May 17, 2019. The current protocol version (1.3.2) was approved on April 5, 2022. The first patient was included August 10, 2019. Recruitment was completed in April 2022. More information about DUMAS, including the progress of the trial and participating centers, can be found on the website (https://dumas-trial.nl/).

## Supplementary Information

**Additional file 1.**

**Additional file 2.**

## Data Availability

The full protocol is available on the DUMAS trial website (https://dumas-trial.nl). Access to the trial dataset and statistical code will be made available upon request to the Principal Investigator. Data may also be shared with non-commercial parties for scientific purposes, including individual patient meta-analyses, and with commercial parties for FDA approval. Consent will be asked specifically for these purposes.

## Abbreviations

DSMB: Data Safety Monitoring Board
DUMAS: Dual thrombolytic therapy with mutant pro-urokinase and small bolus alteplase for ischemic stroke
ICH: Intracranial hemorrhage
IV: Intravenous
m-proUK: Mutant pro-urokinase
MRA: Magnetic resonance angiography
MRI: Magnetic resonance imaging
mRS: Modified Rankin scale
NCCT: Non-contrast computed tomography
NIHSS: National Institutes of Health Stroke Scale
PROBE: Prospective randomized open-label blinded end-point
(S)AE: (Serious) adverse event
WMO: Medical Research Involving Human Subjects Act (in Dutch: Wet Medisch-wetenschappelijk Onderzoek met Mensen)

## References

[1] Wardlaw JM, Murray V, Berge E, del Zoppo GJ (2014). Thrombolysis for acute ischaemic stroke. Cochrane Database Syst Rev.

[2] National Institute of Neurological D, Stroke rt PASSG. Tissue plasminogen activator for acute ischemic stroke. N Engl J Med. 1995;333(24):1581–7. 10.1056/NEJM199512143332401 7477192

[3] Goyal M, Menon BK, van Zwam WH, Dippel DW, Mitchell PJ, Demchuk AM (2016). Endovascular thrombectomy after large-vessel ischaemic stroke: a meta-analysis of individual patient data from five randomised trials. Lancet.

[4] Hansen CK, Christensen A, Ovesen C, Havsteen I, Christensen H (2015). Stroke severity and incidence of acute large vessel occlusions in patients with hyper-acute cerebral ischemia: results from a prospective cohort study based on CT-angiography (CTA). Int J Stroke.

[5] Duvekot MHC, Venema E, Rozeman AD, Moudrous W, Vermeij FH, Biekart M (2021). Comparison of eight prehospital stroke scales to detect intracranial large-vessel occlusion in suspected stroke (PRESTO): a prospective observational study. Lancet Neurol.

[6] del Zoppo GJ, Poeck K, Pessin MS, Wolpert SM, Furlan AJ, Ferbert A (1992). Recombinant tissue plasminogen activator in acute thrombotic and embolic stroke. Ann Neurol.

[7] Gurewich V, Pannell R, Simmons-Byrd A, Sarmientos P, Liu JN, Badylak SF (2006). Thrombolysis vs. bleeding from hemostatic sites by a prourokinase mutant compared with tissue plasminogen activator. J Thromb Haemost.

[8] Liu JN, Liu JX, Liu Bf BF, Sun Z, Zuo JL, Zhang Px PX (2002). Prourokinase mutant that induces highly effective clot lysis without interfering with hemostasis. Circ Res.

[9] Pannell R, Li S, Gurewich V (2015). Highly effective fibrinolysis by a sequential synergistic combination of mini-dose tPA plus low-dose mutant proUK. PLoS One.

[10] Zarich SW, Kowalchuk GJ, Weaver WD, Loscalzo J, Sassower M, Manzo K (1995). Sequential combination thrombolytic therapy for acute myocardial infarction: results of the Pro-Urokinase and t-PA Enhancement of Thrombolysis (PATENT) Trial. J Am Coll Cardiol.

[11] Liu JN, Gurewich V (1992). Fragment E-2 from fibrin substantially enhances pro-urokinase-induced Glu-plasminogen activation. A kinetic study using the plasmin-resistant mutant pro-urokinase Ala-158-rpro-UK. Biochemistry.

[12] Chan AW, Tetzlaff JM, Gotzsche PC, Altman DG, Mann H, Berlin JA (2013). SPIRIT 2013 explanation and elaboration: guidance for protocols of clinical trials. BMJ.

[13] Nederlandse Vereniging voor Neurologie. Herseninfarct en hersenbloeding 2017 [Available from: https://www.neurologie.nl/publiek/beroepsinformatie/richtlijnen/nvn-richtlijnen.

[14] Ma H, Campbell BCV, Parsons MW, Churilov L, Levi CR, Hsu C (2019). Thrombolysis Guided by Perfusion Imaging up to 9 Hours after Onset of Stroke. N Engl J Med.

[15] Bamford J, Sandercock P, Dennis M, Burn J, Warlow C (1991). Classification and natural history of clinically identifiable subtypes of cerebral infarction. Lancet.

[16] Thomalla G, Simonsen CZ, Boutitie F, Andersen G, Berthezene Y, Cheng B (2018). MRI-Guided Thrombolysis for Stroke with Unknown Time of Onset. N Engl J Med.

[17] Bruno A, Shah N, Lin C, Close B, Hess DC, Davis K (2010). Improving modified Rankin Scale assessment with a simplified questionnaire. Stroke.

[18] Wilson JT, Hareendran A, Grant M, Baird T, Schulz UG, Muir KW (2002). Improving the assessment of outcomes in stroke: use of a structured interview to assign grades on the modified Rankin Scale. Stroke.

[19] Kompanje EJO, van Dijck J, Chalos V, van den Berg SA, Janssen PM, Nederkoorn PJ (2020). Informed consent procedures for emergency interventional research in patients with traumatic brain injury and ischaemic stroke. Lancet Neurol.

[20] von Kummer R, Broderick JP, Campbell BC, Demchuk A, Goyal M, Hill MD (2015). The Heidelberg Bleeding Classification: Classification of Bleeding Events After Ischemic Stroke and Reperfusion Therapy. Stroke.

[21] Brott T, Adams HP, Olinger CP, Marler JR, Barsan WG, Biller J (1989). Measurements of Acute Cerebral Infarction - a Clinical Examination Scale. Stroke.

[22] van Swieten JC, Koudstaal PJ, Visser MC, Schouten HJ, van Gijn J (1988). Interobserver agreement for the assessment of handicap in stroke patients. Stroke.

[23] Kaatz S, Ahmad D, Spyropoulos AC, Schulman S (2015). Subcommittee on Control of A. Definition of clinically relevant non-major bleeding in studies of anticoagulants in atrial fibrillation and venous thromboembolic disease in non-surgical patients: communication from the SSC of the ISTH. J Thromb Haemost.

[24] Wahlgren N, Ahmed N, Davalos A, Ford GA, Grond M, Hacke W (2007). Thrombolysis with alteplase for acute ischaemic stroke in the Safe Implementation of Thrombolysis in Stroke-Monitoring Study (SITS-MOST): an observational study. Lancet.

[25] Logallo N, Novotny V, Assmus J, Kvistad CE, Alteheld L, Ronning OM (2017). Tenecteplase versus alteplase for management of acute ischaemic stroke (NOR-TEST): a phase 3, randomised, open-label, blinded endpoint trial. Lancet Neurol.

[26] Huang X, Cheripelli BK, Lloyd SM, Kalladka D, Moreton FC, Siddiqui A (2015). Alteplase versus tenecteplase for thrombolysis after ischaemic stroke (ATTEST): a phase 2, randomised, open-label, blinded endpoint study. Lancet Neurol.

[27] Parsons M, Spratt N, Bivard A, Campbell B, Chung K, Miteff F (2012). A randomized trial of tenecteplase versus alteplase for acute ischemic stroke. N Engl J Med.

[28] Campbell BCV, Mitchell PJ, Churilov L, Yassi N, Kleinig TJ, Dowling RJ (2018). Tenecteplase versus Alteplase before Thrombectomy for Ischemic Stroke. N Engl J Med.

[29] Campbell BC, Mitchell PJ, Churilov L, Yassi N, Kleinig TJ, Yan B, et al. Determining the optimal dose of tenecteplase before endovascular therapy for ischemic stroke (EXTEND-IA TNK Part 2): A multicenter, randomized, controlled study. Int J Stroke. 2020;15(5):567-72. 10.1177/1747493019879652 31564231

[30] Hacke W, Kaste M, Fieschi C, Toni D, Lesaffre E, von Kummer R (1995). Intravenous thrombolysis with recombinant tissue plasminogen activator for acute hemispheric stroke. The European Cooperative Acute Stroke Study (ECASS). JAMA.

[31] Hommel M, Cornu C, Boutitie F, Boissel JP, Multicenter Acute Stroke Trial--Europe Study G (1996). Thrombolytic therapy with streptokinase in acute ischemic stroke. N Engl J Med.

[32] Gusto Angiographic Investigators (1993). The effects of tissue plasminogen activator, streptokinase, or both on coronary-artery patency, ventricular function, and survival after acute myocardial infarction. N Engl J Med.

[33] Hacke W, Kaste M, Fieschi C, von Kummer R, Davalos A, Meier D (1998). Randomised double-blind placebo-controlled trial of thrombolytic therapy with intravenous alteplase in acute ischaemic stroke (ECASS II). Second European-Australasian Acute Stroke Study Investigators. Lancet.

[34] van Kranendonk KR, Treurniet KM, Boers AMM, Berkhemer OA, van den Berg LA, Chalos V (2019). Hemorrhagic transformation is associated with poor functional outcome in patients with acute ischemic stroke due to a large vessel occlusion. J Neurointerv Surg.

[35] Constant Dit Beaufils P, Preterre C, De Gaalon S, Labreuche J, Mazighi M, Di Maria F (2021). Prognosis and risk factors associated with asymptomatic intracranial hemorrhage after endovascular treatment of large vessel occlusion stroke: a prospective multicenter cohort study. Eur J Neurol.

[36] Hao Y, Liu W, Wang H, Zi W, Yang D, Wang W (2019). Prognosis of asymptomatic intracranial hemorrhage after endovascular treatment. J Neurointerv Surg.

