# Supplementary material for: Dual thrombolytic therapy with mutant pro-urokinase and small bolus alteplase for ischemic stroke (DUMAS): study protocol for a multicenter randomized controlled phase II trial
Source: Trials. 2022 Aug 9;23:641. doi: 10.1186/s13063-022-06596-z (PMC9361639; doi:10.1186/s13063-022-06596-z)
Supplement: Supplementary file 1 — Additional file 1. [file 13063_2022_6596_MOESM1_ESM.pdf]

Dual thrombolytic therapy with mutant pro-urokinase and small bolus alteplase for ischemic stroke (DUMAS): study protocol for a multicenter randomized controlled phase II trial

SUPPLEMENTAL MATERIAL

Supplemental File 1. Adaptive design for dose optimization – Design and Simulation report

1. Introduction

Background

This document describes the features of the simulated design, including the statistical models, decision rules, and simulation scenarios as input into the FACTS (Fixed and Adaptive Clinical Trial Simulator) software. A small set of operating characteristics for the simulations is also summarized. The goal of this design is to provide a set of prospectively defined, quantitative decision rules to guide interim analyses in the DUMAS trial. In this design, the DUMAS trial can either proceed to the maximum sample size without any changes, or it can transition to a lower or higher dose of the investigational drug at an interim analysis.

Endpoints

The primary endpoint is freedom from any intracranial hemorrhage (NoICH) after stroke thrombolysis (dichotomous) and is measured within 24 (to 48) hours. The secondary endpoint is clinical improvement within 24 hours (Clin), also dichotomous. A positive outcome is indicated by a value of 1, and a negative outcome (presence of ICH or failure to clinically improve) is indicated by a value of 0. Clinical improvement is defined as improvement of at least 4 points on the National Institute of Health Stroke Scale (NIHSS) at 24 hours compared to baseline, or (near) complete recovery (NIHSS 0 or 1).

Treatment Arms

The trial will enroll up to a maximum of 200 subjects with a discharge diagnosis of ischemic stroke, randomized among 2 arms, including a control arm. We have 1 treatment arm which we label generically by their arm index as: d= 0 (control – standard alteplase dosing), 1 (treatment – investigational thrombolytic regimen – also known as mutant pro-urokinase (mproUK; HisproUK).

2. Statistical modeling

This section describes the statistical modeling used in the design. The modeling is Bayesian in nature.

Final Endpoint Model

The following models are fit separately for the primary and secondary endpoint.

Let Y_i be the primary outcome measured at 24 hours for the ith subject. We model the outcomes as

$$Y_i \sim \text{Bernoulli}(P_{d_i})$$

where P_d is the underlying response rate for arm d. We transform the response rates onto the log-odds scale to allow modeling on a continuous scale:

$$\theta_d = \log\left(\frac{P_d}{1 - P_d}\right).$$

The mean response is modeled independently for each dose as:

$$\theta_0 \sim N(0, 2^2),$$

$$\theta_1 \sim N(0, 2^2).$$

Thus, θ_d for each dose is estimated separately using only data from that dose.

Evaluation of Posterior Estimates

Posterior estimates are independently calculated for each endpoint.

The Bayesian final endpoint model is fitted to the data at each update. The posterior is calculated as:

$$p(\omega|Y) \propto \prod_{i=1}^{n} p\,(y_i|\varphi)p(\varphi)$$

where φ is the set of parameters for the final endpoint model, $p(\varphi)$ is the prior for those parameters, y_i is the final response for each subject, and n is the number of subjects. The posterior is evaluated using MCMC with individual parameters updated by Metropolis Hastings (or Gibbs sampling where possible), using only the y_i data available at the time of the update.

Quantities of Interest

We define a number of quantities that will be tracked and may be used to make decisions during the trial.

Posterior Probabilities

For each dose, we calculate the following quantities from the posterior:

- For the primary endpoint (NoICH), the probability that the mean response on dose d is greater than on control by at least 0.05:

$$Pr(\theta_d - \theta_0 > 0.05)$$

- For the secondary endpoint (Clin), the probability that the mean response on dose d is greater than on control by at least 0.1:

$$Pr(\theta_d - \theta_0 > 0.1)$$

Decision Quantities

Throughout the trial, decisions may be based on the following quantities:

- NoICH endpoint $Pr(\theta_d - \theta_0 > 0.05)$for $dose =$ mproUK
- Clin endpoint: $Pr(\theta_d - \theta_0 > 0.1)$for $dose =$ mproUK

Conventions for Missing Data

At any analysis, some subjects may have missing data for the final endpoint. The missing data could result from the subject dropping out of the study, or because the subject simply has not yet reached the final visit.

If the subject has not yet reached the final visit, the endpoint value is imputed from the estimate of the response for the subjects treatment arm (effectively contributing no information to the update of that estimate).

For any subject whose final endpoint is unknown due to drop out, the final outcome will be multiply imputed from the Bayesian model.

3. Study Design

Timing of Interim Analyses for dose adaptation

The first interim will occur after 60 subjects with a discharge diagnosis of ischemic stroke have data up to 48 hours. Subsequent interims will be conducted after inclusion of every 20 patients with a discharge diagnosis of ischemic stroke and will continue until full accrual. Since interims

are defined by calendar time, the total number of planned interims, I, is random and will depend on the rate at which subjects accrue to the trial. Note that in the initial phase of the trial, mixed quantitative-qualitative review for safety will be carried out by the DSMB, after inclusion of every 10 patients.

<u>Allocation</u>

The trial will enroll 200 subjects with a discharge diagnosis of ischemic stroke that will be randomized to the treatment arms in a fixed ratio. Randomization will occur in blocks of variable sizes.

<u>Criteria for Changing Dose</u>

Changing to a lower dose

For interim 1-I, the trial may transition to a lower dose if BOTH of the following criteria are satisfied:

- $NoICH\ endpoint$: $\Pr(\theta_d - \theta_0 > 0.05) < 0.5$ for $d =$ mproUK
- $Clin\ endpoint$: $\Pr(\theta_d - \theta_0 > 0.1) > 0.5$ for $d =$ mproUK

Changing to a higher dose

For interim 1-I, the trial may transition to a higher dose if all of the following criteria are satisfied:

- $NoICH\ endpoint\ Pr(\theta_d - \theta_0 > 0.05) > 0.5$ for $d =$ mproUK
- $Clin\ endpoint\ Pr(\theta_d - \theta_0 > 0.1) < 0.5$ for $d =$ mproUK

Note that, as per protocol, the results of the interim analysis will be presented to the DSMB, who will advise the chair of the Steering Committee.

Final Evaluation Criteria

At the final analysis, the trial will be considered successful based on the primary endpoint analysis defined in the statistical analysis plan and in the main clinical protocol.

4. Simulation Scenarios

We evaluate the proposed design through trial simulation. We hypothesize several possible underlying truths for the mean response, as well as for trial execution variables such as accrual and dropout. For each of these scenarios, we generate data according to those truths and run through the design as specified above. We repeat this process to create multiple "virtual trials" and we track the behavior of each trial. In this section, we describe the parameters used to generate the virtual subject-level data. Simulations provided below provide what happens until either the trial reaches the maximum sample size without triggering a dose adjustment OR whether a dose change rule is triggered. For example, if a dose increase is recommended at 120 patients, the last 80 patients would be randomized 1:1 to the new dose versus control.

Virtual Subject Response Profiles

We consider 7 profiles for which subject outcomes for the final endpoints are simulated to have response rates as shown in Table 1.

Table 1. Virtual subject response rates

**Scenario**	**NoICH**		**Clin**	
	Control	mproUK	Control	mproUK

BetterBetter	0.8	0.93	0.4	0.6
ICHbetterClinNull	0.8	0.93	0.4	0.4
ICHNullClinBetter	0.8	0.93	0.4	0.6
NullNull	0.8	0.8	0.4	0.4
ICH5betterClinNull	0.8	0.85	0.4	0.4
ICHnullClin10better	0.8	0.8	0.4	0.5
ICH5betterClin10better	0.8	0.85	0.4	0.5

Accrual Profiles

We assume two patients per week for just under 2 years. We simulate the random arrival of subjects into the trial from a Poisson process with the mean weekly rates specified in Table 2. Within each accrual profile, there may be differential recruitment rates over time and across regions. Currently, we simulated only one region for recruitment. Thus, for each region, we specify:

- the mean number of subjects per week at peak accrual,
- the start date (in weeks from the start of the trial),
- whether the region will have a ramp up phase, and if so, when the ramp up will be complete, and
- whether the region will have a ramp down phase, and if so, when the ramp down will begin and when it will be complete. Ramp up and ramp down define simple linear increases and decreases in the mean recruitment rate from the start to the end of the ramp. Thus some simulated trials recruit more quickly than this and some more slowly.

Table 2. Accrual Profiles

Profile Name	Region Index	Peak Rate	Start Week	Ramp Up	Ramp up Complete	Ramp Down	Start Ramp Down	Ramp Down Complete
Acc 1	1	2	0	NA	NA	NA	NA	NA

<u>Dropout Profiles</u>

We assume no dropouts for the purpose of this simulation.

5. Operating Characteristics

For the scenarios described above, we simulate multiple virtual trials and track the behavior of each trial, including the preliminary or final outcome of the trial, the estimated mean response, etc. In this study, the trial will continue with a new dose replacing the initial dose in the event a decision rule is triggered. The results in this section are summarized across all simulated trials for each scenario.

<u>Overall</u>

This section gives a high-level description of the operating characteristics. Table 3 shows the following information per scenario:

- N sim: the number of simulated trials
- E[N]: the expected sample size at the time a dose adaptation is recommended
- Pr(max): the proportion of trials that enroll fully without any interim analysis recommending a dose change
- E[duration]: the expected time until the first dose adaptation trial in weeks.

Table 3. Operating Characteristics Up To First Dose Adaptation

Accrual	Dropout	VSR	N sim	E[N]	Pr(Max)	E[duration]
Acc1	Drop1	BetterBetter	10000	125	0.42	63
Acc1	Drop1	ICHbetterClinNull	10000	68	0.02	35
Acc1	Drop1	ICHNullClinBetter	10000	73	0.04	36
Acc1	Drop1	NullNull	10000	75	0.07	38
Acc1	Drop1	ICH5betterClinNull	10000	71	0.03	36
Acc1	Drop1	ICHnullClin10better	10000	68	0.02	34
Acc1	Drop1	ICH5betterClin10better	10000	74	0.05	37

Trial Outcomes

This section summarizes the outcomes of the simulated trials. For each scenario in Table 4, the columns represent the proportion of simulated trials meeting each of the following definitions:

- Early Dose Increase (EDI): recommended increase in dose at interim analysis
- Early Dose Decrease (EDD): recommended decrease in dose at interim analysis

Table 4. Trial Outcomes Up To First Dose Adaptation

Accrual	Dropout	VSR	EDI	EDD
Acc1	Drop1	BetterBetter	0.30	0.28
Acc1	Drop1	ICHbetterClinNull	0.82	0.16
Acc1	Drop1	ICHNullClinBetter	0.09	0.86
Acc1	Drop1	NullNull	0.32	0.60

Acc1	Drop1	ICH5betterClinNull	0.51	0.45
Acc1	Drop1	ICHnullClin10better	0.21	0.76
Acc1	Drop1	ICH5betterClin10better	0.35	0.59

6. Computational Details

This report reflects the design parameters contained within the TSIdualendpointDec3.facts file. The simulations were run using FACTS (Berry Consultants, LLC, Austin, TX) version 6.2.4. The R software package was used to summarize the simulation output and to create tables for this report.

Supplemental File 2. SPIRIT 2013 Checklist

		Reporting Item	Page and Line Number	Reason if not applicable
**Administrative information**				
Title	#1	Descriptive title identifying the study design, population, interventions, and, if applicable, trial acronym	P 1: L 1-4	
Trial registration	#2a	Trial identifier and registry name. If not yet registered, name of intended registry	P 4: L 6	
Trial registration: data set	#2b	All items from the World Health Organization Trial Registration Data Set	P 4: L 6	
Protocol version	#3	Date and version identifier	P 15: L 18, P 19: L3	
Funding	#4	Sources and types of financial, material, and other support	P 21: L 1-4	
Roles and responsibilities: contributorship	#5a	Names, affiliations, and roles of protocol contributors	P1: L 5-19, P 2: L1-7, P 21: L 5-11	
Roles and responsibilities: sponsor contact information	#5b	Name and contact information for the trial sponsor	P 15: L 4-5	
Roles and responsibilities: sponsor and funder	#5c	Role of study sponsor and funders, if any, in study design; collection, management, analysis, and interpretation of data; writing of the report; and the decision to submit the report for publication, including whether	P 21: L 16-19	

		they will have ultimate authority over any of these activities		
Roles and responsibilities: committees	#5d	Composition, roles, and responsibilities of the coordinating centre, steering committee, endpoint adjudication committee, data management team, and other individuals or groups overseeing the trial, if applicable (see Item 21a for data monitoring committee)	P 15: L 3-11	
**Introduction**				
Background and rationale	#6a	Description of research question and justification for undertaking the trial, including summary of relevant studies (published and unpublished) examining benefits and harms for each intervention	P 5: L 1-23	
Background and rationale: choice of comparators	#6b	Explanation for choice of comparators	P 5: L 1-23	
Objectives	#7	Specific objectives or hypotheses	P 6: L 1-5	
Trial design	#8	Description of trial design including type of trial (eg, parallel group, crossover, factorial, single group), allocation ratio, and framework (eg, superiority, equivalence, non-inferiority, exploratory)	P 6: L 8-17	
**Methods: Participants, interventions, and outcomes**				
Study setting	#9	Description of study settings (eg, community clinic, academic hospital) and list of	P 6: L 17-21	

		countries where data will be collected. Reference to where list of study sites can be obtained		
Eligibility criteria	#10	Inclusion and exclusion criteria for participants. If applicable, eligibility criteria for study centres and individuals who will perform the interventions (eg, surgeons, psychotherapists)	P 7: L 1-16	
Interventions: description	#11a	Interventions for each group with sufficient detail to allow replication, including how and when they will be administered	P 8: L 9-19	
Interventions: modifications	#11b	Criteria for discontinuing or modifying allocated interventions for a given trial participant (eg, drug dose change in response to harms, participant request, or improving / worsening disease)	NA	It is standard procedure to discontinue thrombolytic treatment in case of neurological deterioration until an intracranial hemorrhage is ruled out
Interventions: adherance	#11c	Strategies to improve adherence to intervention protocols, and any procedures for monitoring adherence (eg, drug tablet return; laboratory tests)	NA	No additional action will be taken to monitor this, except standard procedures similar to routine clinical care
Interventions: concomitant care	#11d	Relevant concomitant care and interventions that are permitted or prohibited during the trial	NA	There are no interventions prohibited
Outcomes	#12	Primary, secondary, and other outcomes, including the specific measurement variable (eg, systolic blood pressure), analysis metric (eg, change from baseline, final value, time to event), method of aggregation (eg,	P 10: L 14-22, P 11: L1-2	

		median, proportion), and time point for each outcome. Explanation of the clinical relevance of chosen efficacy and harm outcomes is strongly recommended		
Participant timeline	#13	Time schedule of enrolment, interventions (including any run-ins and washouts), assessments, and visits for participants. A schematic diagram is highly recommended (see Figure)	P 9: L 1-18, P 31: L 1-9	
Sample size	#14	Estimated number of participants needed to achieve study objectives and how it was determined, including clinical and statistical assumptions supporting any sample size calculations	P 14: L 8-21, P 15: L 1-2	
Recruitment	#15	Strategies for achieving adequate participant enrolment to reach target sample size	P 6: L 20-21	
**Methods: Assignment of interventions (for controlled trials)**				
Allocation: sequence generation	#16a	Method of generating the allocation sequence (eg, computer-generated random numbers), and list of any factors for stratification. To reduce predictability of a random sequence, details of any planned restriction (eg, blocking) should be provided in a separate document that is unavailable to those who enrol participants or assign interventions	P 7: L 18-22	

Allocation concealment mechanism	#16b	Mechanism of implementing the allocation sequence (eg, central telephone; sequentially numbered, opaque, sealed envelopes), describing any steps to conceal the sequence until interventions are assigned	P 7: L 18-22	
Allocation: implementation	#16c	Who will generate the allocation sequence, who will enrol participants, and who will assign participants to interventions	P 7: L 18-22, P 8: L 1-8	
Blinding (masking)	#17a	Who will be blinded after assignment to interventions (eg, trial participants, care providers, outcome assessors, data analysts), and how	P 6: L 8-10, P 8: L 1-8	
Blinding (masking): emergency unblinding	#17b	If blinded, circumstances under which unblinding is permissible, and procedure for revealing a participant's allocated intervention during the trial	NA	Open-label trial
**Methods: Data collection, management, and analysis**				
Data collection plan	#18a	Plans for assessment and collection of outcome, baseline, and other trial data, including any related processes to promote data quality (eg, duplicate measurements, training of assessors) and a description of study instruments (eg, questionnaires, laboratory tests) along with their reliability and validity, if known. Reference to where data collection forms can be found, if not in the protocol	P 12: L 8-17	

Data collection plan: retention	#18b	Plans to promote participant retention and complete follow-up, including list of any outcome data to be collected for participants who discontinue or deviate from intervention protocols	P 12: L 8-17	
Data management	#19	Plans for data entry, coding, security, and storage, including any related processes to promote data quality (eg, double data entry; range checks for data values). Reference to where details of data management procedures can be found, if not in the protocol	P 12: L 8-17	
Statistics: outcomes	#20a	Statistical methods for analysing primary and secondary outcomes. Reference to where other details of the statistical analysis plan can be found, if not in the protocol	P 12: L 18-22, P 13: L 1-22, P 14: L 1-7, SAP is available on the website (https://dumas-trial.nl/)	
Statistics: additional analyses	#20b	Methods for any additional analyses (eg, subgroup and adjusted analyses)	P 12: L 18-22, P 13: L 1-22, P 14: L 1-7, SAP is available on the website (https://dumas-trial.nl/)	
Statistics: analysis population and missing data	#20c	Definition of analysis population relating to protocol non-adherence (eg, as randomised analysis), and any statistical methods to handle missing data (eg, multiple imputation)	P 12: L 18-22, P 13: L 1-22, P 14: L 1-7, SAP is available on the website	

			(https://dumas-trial.nl/)	
**Methods: Monitoring**				
Data monitoring: formal committee	#21a	Composition of data monitoring committee (DMC); summary of its role and reporting structure; statement of whether it is independent from the sponsor and competing interests; and reference to where further details about its charter can be found, if not in the protocol. Alternatively, an explanation of why a DMC is not needed	P 11: L 3-22, P 12: L 1-6	
Data monitoring: interim analysis	#21b	Description of any interim analyses and stopping guidelines, including who will have access to these interim results and make the final decision to terminate the trial	P 11: L 3-22, P 12: L 1-6	
Harms	#22	Plans for collecting, assessing, reporting, and managing solicited and spontaneously reported adverse events and other unintended effects of trial interventions or trial conduct	P 11: L 3-22, P 12: L 1-6	
Auditing	#23	Frequency and procedures for auditing trial conduct, if any, and whether the process will be independent from investigators and the sponsor	P 12: L 8-9	
**Ethics and dissemination**				

Research ethics approval	#24	Plans for seeking research ethics committee / institutional review board (REC / IRB) approval	P 15: L 12-19	
Protocol amendments	#25	Plans for communicating important protocol modifications (eg, changes to eligibility criteria, outcomes, analyses) to relevant parties (eg, investigators, REC / IRBs, trial participants, trial registries, journals, regulators)	P 15: L 12-19	
Consent or assent	#26a	Who will obtain informed consent or assent from potential trial participants or authorised surrogates, and how (see Item 32)	P 9: L 19-22, P 10: L 1-13	
Consent or assent: ancillary studies	#26b	Additional consent provisions for collection and use of participant data and biological specimens in ancillary studies, if applicable	P 9: L 19-22, P 10: L 1-13, P 19: L 19-20, P 20: L 1-2	
Confidentiality	#27	How personal information about potential and enrolled participants will be collected, shared, and maintained in order to protect confidentiality before, during, and after the trial	P 12: L 9-13	
Declaration of interests	#28	Financial and other competing interests for principal investigators for the overall trial and each study site	P 20: L 11-19	
Data access	#29	Statement of who will have access to the final trial dataset, and disclosure of contractual agreements that limit such access for investigators	P 20: L 5-10	

Ancillary and post trial care	#30	Provisions, if any, for ancillary and post-trial care, and for compensation to those who suffer harm from trial participation	NA	No special actions, other than regular trial insurance as provided by the METC after approval
Dissemination policy: trial results	#31a	Plans for investigators and sponsor to communicate trial results to participants, healthcare professionals, the public, and other relevant groups (eg, via publication, reporting in results databases, or other data sharing arrangements), including any publication restrictions	P 19: L 19-20, P 20: L 1-2	
Dissemination policy: authorship	#31b	Authorship eligibility guidelines and any intended use of professional writers	NA	Manuscript will be written by the authors
Dissemination policy: reproducible research	#31c	Plans, if any, for granting public access to the full protocol, participant-level dataset, and statistical code	P 20: L 6-10	
**Appendices**				
Informed consent materials	#32	Model consent form and other related documentation given to participants and authorised surrogates	Available on the website (https://dumas-trial.nl/)	
Biological specimens	#33	Plans for collection, laboratory evaluation, and storage of biological specimens for genetic or molecular analysis in the current trial and for future use in ancillary studies, if applicable	P 9: L 1-18	
